# Learnings from the first AI-enabled skin cancer device for primary care authorized by FDA

**DOI:** 10.1038/s41746-024-01161-1

**Published:** 2024-06-15

**Authors:** Kaushik P. Venkatesh, Kushal T. Kadakia, Stephen Gilbert

**Affiliations:** 1 Harvard Medical School, Boston, MA USA; 2 Else Kröner Fresenius Center for Digital Health, TUD Dresden University of Technology, Dresden, Germany

**Keywords:** Health policy, Health services

## Abstract

The U.S. Food and Drug Administration’s (FDA) recent authorization of DermaSensor, an AI-enabled device for skin cancer detection in primary care, marks a pivotal moment in digital health innovation. Clinically, the authorization of the first AI-enabled device for use by non-specialists for detecting skin cancer reinforces the feasibility of digital health technologies to bridge gaps in access and expertise in medical practice. The authorization also establishes a new regulatory precedent for FDA authorization of medical devices incorporating AI and machine learning (ML) technologies within dermatology. Together, this article uses the DermaSensor authorization to examine the clinical evidence and regulatory implications of emerging AI-enabled technologies in dermatology.

On January 17, 2024, the U.S. Food and Drug Administration (FDA) authorized DermaSensor, the first artificial intelligence (AI)-enabled medical device intended for use in the primary care setting for skin cancer detection^1^. The device, which was already authorized for use in the European Union and Australia, establishes a new regulatory precedent in the U.S. for medical device innovation as the first AI-enabled dermatologic device indicated for use by non-specialist. However, the approval also raises questions about the need for continuous evidence generation and modern approaches to regulation.

Clinical diagnosis in dermatology is highly reliant on visual assessment. Differentiating between “normal” and “abnormal” skin lesions—which in turn determines the need for further diagnostic studies and intervention—depends on a dermatologist’s pattern recognition skills, which compound over the course of a clinical career. Performing nuanced visual analysis requires years of training, posing both a challenge for non-specialists^2^ and an opportunity for innovation through AI/ML pattern recognition. Skin cancer—including squamous cell carcinoma, basal cell carcinoma, and melanoma—is the most common malignancy worldwide^3^ and is primarily screened through gross assessment and dermatoscopy prior to diagnosis by biopsy or excision. Primary care physicians often screen patients for concerning lesions prior to referral to dermatology^2,3^. With this context, DermaSensor and similar PCP-targeted AI-enabled devices provide critical opportunities to impact the broader healthcare system by bringing specialist care into the primary care office. This article uses the DermaSensor authorization to examine the clinical evidence and regulatory implications of emerging AI-enabled technologies in dermatology.

## Regulatory landscape and new horizons

FDA’s framework for medical device regulation was established when most products were hardware-based technologies. However, many devices today incorporate some elements of software technology, including AI and machine learning (ML), with 171 AI/ML devices authorized by FDA as of October 2023. Some products, such as DermaSensor, are physical devices that rely on AI/ML software for functionality, and are termed “Software in a Medical Device” (SiMD)^4,5^. Other products, such Digital Diagnostic’s autonomous diabetic retinopathy software^6^, are primarily software-based products, and are termed “Software as a Medical Device” (SaMD)”^4^.

In a 2021 Action Plan, FDA clarified its intent to continue regulating such devices using its existing review pathways, while also developing new regulatory processes for issues unique to devices incorporating software (e.g., change control planning, mitigation of algorithmic bias)^7^. These review pathways consist of premarket clearance (510(k)), De Novo classification, and premarket approval (PMA). Review pathways are selected based on the potential risk posed by a device, which in turn determines requirements for pre- and post-market evidence generation.

The first dermatology AI device to gain FDA authorization was MelaFind (multispectral spectroscopy) approved in 2017 via the PMA pathway (Table 1)^8^. This pathway is reserved for high-risk devices and requires manufacturers submit clinical evidence of safety and effectiveness (e.g., evidence generated from a clinical trial). MelaFind’s indication for use was limited to dermatologists and melanoma detection (Table 2). However, the device was discontinued due to low specificity (10%) leading to unnecessary biopsies, narrow use cases, poor integrability into workflow, and limited coverage^9^. The next dermatology AI device, Nevisense (electrical impedance spectroscopy), was also authorized under the PMA pathway in 2017 for use by dermatologists alone for melanoma detection (sensitivity 96%, specificity 34%), and remains on the market as of 2024^10^.

**Table 1:** Comparison of pathways to FDA authorization for medical devices, including AI-enabled dermatology devices

Category	PMA	De Novo	510(k)
AI-enabled dermatology device	NeviSense & MelaFind	DermaSensor	None
Risk classification	High risk	Low-moderate risk	Low-moderate risk
Qualifications for pathway	Novel, life-sustaining without predicate	Novel device without predicate	Substantially equivalent predicate device
Required data to submit to FDA	Both non-clinical and clinical evidence of safety and effectiveness	Non-clinical and sometimes clinical data demonstrating the device’s safety and effectiveness	Demonstration that the new device is similar to a previously-authorized device; primarily relies on non-clinical evidence of device performance, but may in some cases include clinical data of safety and effectiveness
Review time and cost	Slower (<180 days) and more expensive due to requirement for clinical data	Generally faster (<150 days)	Fastest review (<90 days)
Post-market	Often required to complete a post-approval study, with requirements for regular data reports for FDA	May be subject to requirements for postmarket evidence generation	Rarely subjected to formal postmarket surveillance

**Table 2:** Comparison of pivotal study sizes and primary endpoints among FDA-authorized AI-enabled medical devices for skin cancer detection

Device	Imaging technology	Indication	Patient *n* (Lesions *n*)	Sensitivity	Specificity	Lesions of Fitzpatrick V/VI (%)
Melafind^21^	Multispectral spectroscopy	Disease: Melanoma User: Dermatologist	1383 (1631)	98.3%	9.9%	9 (0.6%)
NeviSense^22^	Electrical impedance spectroscopy	Disease: Melanoma User: Dermatologist	1951 (2416)	97.0%	31.3%	29 (1.6%)
DermaSensor^13^	Elastic scattering spectroscopy	Disease: Melanoma, squamous cell carcinoma, and basal cell carcinoma in patients >40 years User: Primary care physician	1005 (1579)	96.6%	21.0%	128 (12.7%)^a^

^a^Publicly-available FDA documents for DermaSensor report data by patients rather than lesions.

In contrast to MelaFind and Nevisense, DermaSensor (elastic scattering spectroscopy) was reviewed under FDA’s De Novo pathway. This pathway is intended for novel devices of low-to-moderate risk for which the manufacturer or FDA lack similar, authorized devices to compare to. Consequently, in addition to enabling a new product (DermaSensor) to progress to market, De Novo authorization also establishes a new product classification code which future dermatology devices can use as regulatory precedent to gain FDA authorization via the 510(k) pathway—a unique regulatory development compared to the PMA approach of AI-enabled dermatology devices to date (Table 2)^11^. While 510(k) is the predominant route to market entry for all medical devices–including AI/ML-enabled devices—the pathway has raised concerns about the adequacy of its evidentiary requirements for assessing the safety of new digital health tools^12^.

## Examining the evidence for the DermaSensor authorization

FDA’s authorization of DermaSensor broke new clinical ground, as the device is indicated for use by non-dermatologist physicians to evaluate skin lesions raising concern for melanoma, basal cell carcinoma, and squamous cell carcinoma in patients 40 years or older. Evidence supporting the authorization was generated from three studies: a pivotal trial, a supplemental validation study for melanoma, and a clinical utility study^13^.

The pivotal study, titled DERM-SUCCESS, was conducted on 1579 lesions of 1005 patients from 22 primary care centers; the supplemental validation study, DERM-ASSESS, included 311 patients with 440 lesions evaluated by dermatologists from 10 sites. The device’s output was used in addition to traditional clinical history and examination to decide whether suspicious lesions should be monitored versus further evaluated (dermatologist referral and/or biopsy). DERM-SUCCESS reported 95.5% device sensitivity compared with 83% for primary care physicians (PCPs), an NPV (negative result by device confirmed by biopsy) of 96.6%, and non-inferiority to dermatologist sensitivity of 90%. However, specificity was low at 20.7%.

Notably, FDA’s authorization specifies that DermaSensor is approved for use by “physicians who are not dermatologists,” with the manufacturer identifying PCPs as a target population^14^. The clinical utility study of 108 PCPs and >10,000 lesions showed that PCP device use increased management sensitivity (91.4% vs. 82.0%) and diagnostic sensitivity (81.7% vs. 71.1%) and decreased false negative referrals by half (8.6% from 18%). However, there was a statistically significant decrease in specificity (44.2% to 32.4%) for referrals.

Taken together, the results from DermaSensor’s premarket clinical studies demonstrate the device’s capability for sensitive detection and application in the primary care setting. However, DermaSensor also, illustrates that issues with specificity (which affected MelaFind and Nevisense) remain a challenge for AI-enabled medical devices in dermatology, carrying the risk of potentially unnecessary referrals that may result in biopsies and further costs.

## Clinical implications for access and quality

DermaSensor provides new horizons for dermatologic care by extending the diagnostic capacity of PCPs for skin cancer; a key differentiator from previous FDA-authorized AI-enabled medical devices in dermatology. Using DermaSensor could strengthen the diagnostic abilities of PCPs who normally refer such cases, and could therefore meaningfully address access limitations in dermatology, which affect more than a third of patients^15^ with an average wait time of 35 days^16^.

There are analogous situations of the introduction of novel AI-based diagnostic devices into primary care from other medical disciplines. For example, FDA has authorized AI-enabled devices for autonomous diabetic retinopathy screenings for use in primary care settings^17^. This technology-enabled shift toward greater “specialist” diagnosis by PCPs raises important questions about the expanding scope of generalist clinical practice. For DermaSensor, an important question is whether the device will improve PCP diagnostic performance enough to warrant deferral of dermatologist evaluation. Additionally, realizing the aspiration of improved diagnostic access is also dependent on the uptake of new technologies among PCPs serving the populations with the greatest needs. For example, rural patients face the longest wait-times and greatest shortages of dermatologists^18^; however, only 5% of PCPs in DERM-SUCCESS were considered rural. Device deployment among providers in medically underserved areas warrants further postmarket research.

DermaSensor’s authorization also comes with notable regulatory conditions. FDA has imposed requirements for post-market performance testing in underrepresented populations, as notably 97.1% of patients in the pivotal DERM-SUCCESS trial were of White race and only 13% were from the most pigmented skin types (Fitzpatrick V/VI). While improving clinical trial diversity is a priority across all FDA-regulated products, the implications are particularly salient in dermatology, where the lack of darker skin tones in images used to train AI models have resulted in poorer diagnostic performance on darker skin phenotypes^19^. The lack of representation risks re-entrenching diagnostic biases for patients who are already systematically and historically marginalized in dermatology care^20^. In addition to investigating equitable performance across patient populations, FDA should also monitor DermaSensor’s real-world performance with particular attention to diagnostic specificity and the risk of triggering unnecessary care cascades through false positives; an issue that contributed to the discontinuation of MelaFind.

### The path forward

DermaSensor’s authorization is the latest in the ongoing wave of AI-enabled medical devices to gain regulatory approval in the U.S. and beyond. Understanding the opportunities and limitations of these technologies is critical to their safe and effective deployment in real-world clinical practice. The approach taken by FDA is notable for its equity-focused lens to ensure alignment between new product development and population health needs via post-market evidence generation. This approach should be proactively implemented in the regulatory approach to AI-enabled devices more broadly, and ideally at the pre-market stage. Taken together, while the short-term impact of DermaSensor’s authorization is the addition of a new AI-enabled specialty tool in the primary care toolkit, the device’s long-term legacy may be a milestone for the regulation of AI-enabled medical devices.

### Reporting summary

Further information on research design is available in the Nature Research Reporting Summary linked to this article.

### Supplementary information

Reporting Summary
